# Laparoscopic radical prostatectomy

**DOI:** 10.4103/0972-9941.19267

**Published:** 2005-10

**Authors:** Michael Lipke, Chandru P. Sundaram

**Affiliations:** Department of Urology, Indiana University School of Medicine, Indianapolis, Indiana, USA

**Keywords:** prostate cancer, laparoscopic prostatectomy, radical

## Abstract

Millions of men are diagnosed annually with prostate cancer worldwide. With the advent of PSA screening, there has been a shift in the detection of early prostate cancer, and there are increased numbers of men with asymptomatic, organ confined disease. Laparoscopic radical prostatectomy is the latest, well accepted treatment that patients can select. We review the surgical technique, and oncologic and functional outcomes of the most current, large series of laparoscopic radical prostatectomy published in English.

Positive margin rates range from 2.1–6.9% for pT2a, 9.9–20.6% for pT2b, 24.5–42.3% for pT3a, and 22.6–54.5% for pT3b. Potency rates after bilateral nerve sparing laparoscopic radical prostatectomy range from 47.1 to 67%. Continence rates at 12 months range from 83.6 to 92%.

Several treatment options exist for the treatment of localized prostate cancer. Laparoscopic radical prostatectomy is the most novel accepted technique and was introduced in the 1990s.[1,2] It aims to replicate the results that have been obtained by open radical retropubic prostatectomy while reducing the morbidity associated with surgery. Since its introduction, laparoscopic radical prostatectomy has undergone numerous modifications in surgical technique, including approach (transperitoneal[2] vs extraperitoneal[3]), anterior[4] and posterior[2] dissection, ascending[5] and descending[4] dissection, and most notably, robotic-assisted. As with all new techniques, the learning curve must be surmounted to become proficient, and modifications are made to improve outcome. Much of the current data for laparoscopic prostatectomy includes outcomes from expert surgeons'early experience.[6] Oncologic survival data for conventional treatments of localized prostate cancer has reached maturity, and routine 5–15 year follow-up is available.[7–11] No similar long term data exists for laparoscopic prostatectomy. Potency, continence and pathologic staging data, however, are usually apparent within 18 months of follow-up, and ample data is available. We reviewed the most recently available published data of large series laparoscopic prostatectomy from centers of excellence. Robotic assistance is not included in this review.

## SURGICAL TECHNIQUE

### Open

Conventional open radical retropubic prostatectomy is performed extraperitoneally, and usually in a retrograde fashion after dissection of the urethra.[1–2] The urethro-vesical anastomosis sutures are interrupted, and secured without direct vision.

### Laparoscopic

#### Extraperitoneal vs Transperitoneal:

**Figure 1:**
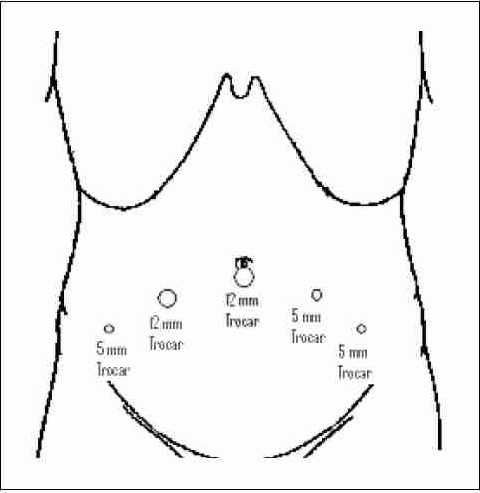
Port placement for laparoscopic radical prostatectomy

The initial technique developed in France was transperitoneal to accommodate for sufficient working space for the trocars, and to access the seminal vesicles. [2] Typically, 5 trocars are placed in a fan array (Figure 1): a 10 mm umbilical trocar for the laparoscope, another 10 mm trocar is placed on the right side on the lateral edge of the rectus muscle, a 5 mm trocar on the left lateral edge of the rectus abdominis muscle, and two more 5 mm trocars are inserted approximately 2 cm medial and superior to the anterior superior iliac spines. Pneumoperitoneum is usually obtained using a Veress needle. The space of Retzius is developed via a transperitoneal approach and the bladder neck is divided to gain access to the ampulla of the vas and seminal vesicles. This step initially involves an inverted U shaped peritoneal incision from one medial umbilical ligament to the other. Further modification of access technique allowed similar exposure of the space of Retzius via an extraperitoneal approach while allowing similar trocar access. A balloon dilator is placed through the umbilical incision and a preperitoneal space is created anterior to the bladder. After creation of the working space, a blunt tipped balloon trocar is placed to prevent a gas leak. At this point, the additional trocars can be placed in the same location, yet not traversing the peritoneal cavity. Many experienced laparoscopists are not encumbered by the diminished working space.

Theoretical advantages for the extraperitoneal approach include: less postoperative ileus, avoiding abdominal organs from moving into the operative field, and eliminating urine spilling into the abdominal cavity. Abdominal organ injury can still occur if the trocars pass inadvertenly through the peritoneum, and these injuries may not be readily apparent. Operative times have been shown to be less in the extraperitoneal approach.[13] The transperitoneal approach has a decreased risk of post operative lymphocele if pelvic lymphadenectomy is performed.[14] Either technique is acceptable, and surgeons should proceed with the approach that they are most familiar with.

#### Descending vs ascending:

Conventional open radical retropubic prostatectomy is performed in an ascending fashion with initial transection of the urethra, cephalad dissection of the prostate off the rectum, dissection of the ampulla of the vas and seminal vesicles, and the transection of the bladder neck (the latter two steps may be reversed). The initial development of the laparoscopic technique was performed in a descending fashion via a posterior approach with dissection of the seminal vesicles and vas via the vesicorectal pouch.[2] At this point, the space of Retzius is entered, the endopelvic fascia incised, and dorsal venous complex ligated. The bladder neck is then transected, and the previously dissected seminal vesicles and ampulla are encountered. Today, most surgeons will approach anteriorly[4] with dissection of the seminal vesicles after transecting the bladder neck, thereby avoiding retrovesical dissection and possible ureteral injury.[15] Once the seminal vesicles are dissected and the ampulla of the vas transected, the prostate is dissected off the rectum in a descending approach. The vas deferens and the seminal vesicle are held anteriorly while the prostatic pedicles are controlled with bipolar coagulation or with locking polymer clips. The neurovascular bundles are then carefully dissected away from the posterolateral surface of the prostate. The prostate is then only attached at the apex. The dorsal venous complex is divided and the urethra transected. The rectourethralis muscle is then divided. This step usually completes the prostatic dissection. The neurovascular bundles are safeguarded during the apical dissection.

In order to maintain similarity to the open technique, some surgeons prefer to perform the dissection in an ascending approach (Heilbronn Technique)[5] as mentioned previously with the open technique.

#### Nerve Sparing vs Non Nerve Sparing:

Preservation of the cavernosal nerves lying on the posterolateral aspect of the prostate is crucial for maintenance of potency.[16] Large volume, high grade cancer may preclude preservation of the nerves and pre-operative potency may determine whether a wide dissection is indicated. In either scenario, the dissection can be performed laparoscopically and may be facilitated by the improved visualization provided by magnification.[17] Important considerations during the dissection of the neurovascular bundles include limited use of thermal energy to preserve the nerves.

### Vesicourethral anastomosis

There is considerable variability in this step. Some surgeons prefer to place interrupted sutures [5,15] while others perform a running suture anastomosis.[4] An interrupted suturing requires more knot tying, and may be more time consuming. Many surgeons today perform the anastomosis in a running fashion using 2 pretied sutures. The anastomosis starts posteriorly, leaving the knot external to the bladder neck, and finishes anteriorly,where only one intracorporeal knot is tied. Bladder neck reconstruction may be required if the opening is too large.

#### Lymph node dissection:

If indicated by preoperative PSA, grade and stage, laparoscopic pelvic lymphadenectomy is easily performed without adding significant time or morbidity to the surgery.

#### Specimen retrieval:

After the anastomosis is completed, the prostate is placed in a disposable sac and removed via the larger trocar site, which usually requires a few centimeters extension of the incision for extracting the specimen.

## ONCOLOGIC DATA

The primary goal of radical prostatectomy is surgical cure of prostate cancer. Important prognostic factors that influence oncologic results include: PSA, clinical and pathologic stages, margin status, and lymph node involvement. Because of the indolent nature of most prostate cancer, long-term follow-up is needed to determine disease-free survival. PSA failure may occur several years after radical prostatectomy. Following laparoscopic radical prostatectomy, only up to 5 year data on oncologic results has been published. [6]

In one of the larger series of laparoscopic prostatectomies, Stolzenburg et al reviewed both the functional and oncologic outcomes in 700 patients.[13] Fifty five percent of patients were pT2, and 44% were pT3, with positive margins in 10.8 % and 31.2 % respectively. Only 8 patients had PSA recurrence at 12 months in their series, however, many patients with high risk disease had immediate adjuvant therapy. In a review of 230 patients, Anastasidis et al reported a 26.5 % positive margin rate.[18] In a smaller sample size, Gill et al reported a 15% positive margin rate in their most recent patient cohort. [19]

Rassweiller et al reported the 5 year data from their initial 500 laparoscopic radical prostatectomies. [6] Eighty three percent of patients underwent pelvic lymphadenectomy with a 1.4% positivity rate. Nineteen percent of tumors were pT2a, 40.2% were pT2b, 21.4 % were pT3a, 12.8% were pT3b, and 3.8% were pT4. The overall positive margin rate was 19.0%, with 48% occurring at the apex, and 16.8% at the bladder neck.

Patients with high risk disease were treated with adjuvant therapy (22.3%), and 11.8% of all patients had PSA relapse at a mean of 20.8 months. The incidence of PSA recurrence was 3.2% for pT2a, 6.5% for pT2b, 15.9% for pT3a, and 23.9% for pT3b/4. Overall PSA progression-free rates were 83.0% and 73.1% at 3 and 5 years, but were 95.2% and 89.5% for pT2 tumors.

In the largest series, Guilloneau et al reviewed their first 1000 cases at the Montsouris Institute.[20] Pelvic lymphadenectomy was performed in 21.6% of patients with a 97.2% negativity rate. Pathologic tumor stage and corresponding positive margin status were as follows: pT2a 20.3% and 6.9%, pT2b 57.2% and 18.0%. pT3a 14.2% and 30%, and pT3b 7.7% and 32%. The 3 year progression free survival was 91.8% for pT2a, 88% for pT2b, 77% for pT3a, and 44% for pT3b. No port site metastases occurred, and the location of positive surgical margins was apical in 50%, posterolateral in 30%, and basilar in 20%.

## FUNCTIONAL DATA

In countries with established screening programs, most men diagnosed with prostate cancer are asymptomatic (cT1c), yet all forms of curative treatment have significant morbidity. Preservation of potency and continence are two of the most important non-oncologic concerns for patients.

### Potency

When indicated, nerve-sparing laparoscopic radical prostatectomy can be performed without compromising oncologic results.[21] In the last 656 patients treated at the Montsouris Institute, nerves were spared bilaterally in 55.9% and unilaterally in 16%.

Link et al reviewed the quality of life changes after laparoscopic radical prostatectomy.[22] Of 122 patients studied, 48.4% underwent bilateral nerve-sparing and 32.8% unilateral. Using validated questionnaires, sexual function scores at 12 months post-op were at 64% of pre-op values. Of the 50 patients who were potent pre-op and underwent bilateral nerve sparing, 78.9% were engaging in sexual intercourse at 12 months.

In the earlier mentioned review by Anastasiadis et al, of 230 patients, 33% underwent bilateral and 14.3% underwent unilateral nerve sparing surgeries.[18] Patients under 60 years of age who underwent bilateral nerve sparing laparoscopic radical prostatectomy had potency rates of 81% at one year, compared to 72% for those who underwent open radical prostatectomy. For all age groups, unilateral and bilateral nerve sparing resulted in 46% and 53% potency rates at one year.

Of the 500 patients reviewed by Rassweiller et al[6], 13.6% underwent unilateral nerve sparing and 8.2% underwent bilateral nerve sparing. Potency rates for bilateral nerve sparing at 12 months was 67%. Of the 700 patients in their series, Stolzenberg et al[13] preserved nerves unilaterally and bilaterally in 16.3% and 10.1%, respectively. At 6 months, potency rates were 12.1 % and 47.1%, respectively.

**Table 1:** Oncologic and Functional results from large Laparoscopic prostatectomy Series

Author	patients	age	OR time (+LN/−LN)	Approach	PMpT 2a	PMpT 2b	PMpT 2c	PMpT 3a	PMpT 3b	potent (Bil/Uni)	continent (3/mo/6/12)	catheter time (days)	EBL (ml)	Transfusion Rate
Guillonneau (20)	1000	63		TLRP	6.9	18		30	32						
Rassweiler (6)+(25)	500	64	246	TLRP	2.1	9.9		25.2	42	67/-	51/70/83.6			
Stolzenburg (13)	700	63.4	170/115	ELRP	2.2	14.8	13.1	24.5	50.6	47.1/13.3	73.8/83.8/92	6.2	220	0.9
Ruiz (26)	165	64.1	260/240	TLRP	4.5	14.8		36.4	54.5			5.1	678	1.2
	165	62.9	235/210	ELRP	4.8	20.6		42.3	50			6.6	803	5.4
Rozet (27)	600	62	173	ELRP	6.7	14.9	16.5	26.9	22.6	64/-	-/-/91	7.6	380	1.2

Key: LN: Lymph Node; TLRP- Transperitoneal Laparoscopic Prostatectomy; ELRP- Extraperitoneal Laparoscopic Prostatectomy; PM- Positive Margin

### Continence

In an early series by Abbou, at one year follow-up, 78.4% of patients wore no pads, and 100% wore 1 pad.[23] In the series by Gill et al[19], 94% of patients were continent at 6 months. In Rassweiller's series, continence rates with no pads at 12 and 24 months were 83.6% and 97.7%, respectively.[6] Link et al reported that up to 1 pad per day was used in 51%, 89.9%, and 93.4% at 3, 6, and 12 months, respectively.[22]

## OPERATIVE AND PERIOPERATIVE

Brown et al compared their experience with extraperitoneal and transperitoneal laparoscopic radical prostatectomy, and their operative times were 191 and 197 min, respectively.[24] Pelvic lymphadenectomy and herniorraphy were performed in 30.7% and 10.9% of cases, respectively. In Gill's first 50 cases, average operative time was 5.4 hours, and it currently ranges from 2–3 hours.[19] Rassweiller et al performed laparoscopic radical prostatectomy with lymphadenectomy in 218 min.[6]

## CONCLUSION

Laparoscopic radical prostatectomy has come a long way in just a few short years. With continued laparoscopic experience and modification in technique, laparoscopic radical prostatectomy has proven equal to open radical prostatectomy. Surgeons expert at open prostatectomy have reached the limit of perfecting open radical prostatectomy. Laparoscopic prostatectomy has barely reached adolescence, and already rivals open prostatectomy in surgical outcome. Once it reaches its full maturity, laparoscopic radical prostatectomy may surpass its predecessor.
